# Correction to “Surgical Outcomes of Video‐Assisted Thoracic Surgery Combined With Computed Tomography‐Guided Microwave Ablation for Lung Cancer Presenting as Multiple Ground‐Glass Opacities: A 5‐Year Retrospective Cohort Study”

**DOI:** 10.1111/1759-7714.70142

**Published:** 2025-07-30

B. Huang, C. Zhan, P. Yu, et al., “Surgical Outcomes of Video‐Assisted Thoracic Surgery Combined With Computed Tomography‐Guided Microwave Ablation for Lung Cancer Presenting as Multiple Ground‐Glass Opacities: A 5‐Year Retrospective Cohort Study,” *Thoracic Cancer* 16, no. 14 (2025): e70120, https://doi.org/10.1111/1759‐7714.70120.

In paragraph 1 of the “2.1 Study Design” section, the text “ChiCTR×××××t.” was incorrect. This should have read: “ChiCTR1900022159.”

In the “Ethics Statement” section, the text “This retrospective study was conducted in the Thoracic Surgery Department of ×× Hospital, China. This study was reviewed and approved by the Ethics Committee of ×× Hospital. All patients included in the study provided written consent to publish the related research results. Ethical approval for this study (Ethical Committee 2019‐×××) was provided by the Ethics Committee of ×× Hospital, Nanjing, China, on 28 February 2019.” was incorrect. This should have read: This study was reviewed and approved by the Ethics Committee of Jiangsu Cancer Hospital. All patients included in the study provided written consent to publish the related research results. Ethical approval for this study (Ethical Committee 2019‐022) was provided by the Ethics Committee of Jiangsu Cancer Hospital, Nanjing, China, on 28 February 2019.

We apologize for this error.

